# Functional and structural outcome after vitrectomy combined with subretinal rtPA Injection with or without additional intravitreal Bevacizumab injection for submacular hemorrhages

**DOI:** 10.1371/journal.pone.0250587

**Published:** 2021-04-30

**Authors:** Annekatrin Rickmann, Lina R. Paez, Maria della Volpe Waizel, Lukas Bisorca-Gassendorf, André Schulz, Anne-Cecile Vandebroek, Peter Szurman, Kai Januschowski

**Affiliations:** 1 Depatment of Ophthalmology, Knappschaft Hospital Saar, Sulzbach/Saar, Germany; 2 Department of Ophthalmology, University of Basel, Basel, Switzerland; 3 Depatment of Ophthalmology, University Eye Clinic Tuebingen, Tuebingen, Germany; National Yang-Ming University Hospital, TAIWAN

## Abstract

**Background:**

To analyze the functional and anatomical outcome after vitrectomy with subretinal rtPA (recombinant tissue plasminogen activator) combined with or without an intravitreal Bevacizumab injection.

**Patients and methods:**

Retrospective, consecutive case series of 31 pseudophakic patients with submacular hemorrhage (SMH) due to neovascular age-related macular degeneration (AMD) treated with vitrectomy, subretinal rtPA and pneumatic air displacement with or without an additional intravitreal Bevacizumab injection. The primary endpoints were best-corrected visual acuity (BCVA), and central macular thickness (CMT) measured by SD‑OCT. The secondary endpoint was a displacement of hemorrhage from the subretinal space three months after surgery.

**Results:**

31 eyes of 31 patients were treated with vitrectomy and subretinal rtPA. 17/31 were treated simultaneously with an intravitreal Bevacizumab injection (group +B) and 14/31 without (group -B). The mean visual acuity improved significantly in both groups (from 1.37±0.39 to 1.03±0.57 logMAR in +B and from 1.48±0.48 to 1.01±0.38 logMAR in group –B, p<0.05). The mean CMT decreased in group +B from 607±179 μm to 424±205 μm (p = 0.2) and in group –B from 722±216 μm to 460±202 μm (p<0.05). A central displacement of the hemorrhage could be achieved in 47% in group +B, whereas in group -B displacement could be achieved in 50% (p = 0.44).

**Conclusions:**

Vitrectomy with subretinal rtPA injection and air tamponade with or without simultaneous intravitreal Bevacizumab injection displaces SMH and improves BCVA effectively. In comparison, the postoperative outcome is comparable regardless of whether or not intravitreal bevacizumab is applied simultaneously.

## Introduction

Submacular hemorrhage (SMH) is a rare but severe complication of choroidal neovascularization (CNV) in age-related macular degeneration (AMD). If they remain untreated, SMH leads to rapid and profound loss of visual acuity [1–3] and can cause severe degeneration of photoreceptors due to iron toxicity, fibrin meshwork contraction, reduced nutrient flux, with subsequent macular scarring, as well as damage of the retinal pigment epithelium (RPE) [4–6].

The main goal of any intervention is the displacement of the toxic blood clot away from the fovea without inducing too much iatrogenic complications. Several surgical techniques have been proposed to displace SMH with variable success [7]. To minimise retinal manipulation while allowing maximum contact between tPA and SMH, followed by pneumatic displacement, subretinal injection of tPA is performed during vitrectomy [8, 9]. As it is also important to treat the underlying cause of the hemorrhage, the CNV in AMD, results improved when treatments were combined with anti-VEGF therapy (anti–vascular endothelial growth factor) [10–12]. However, there is still no consensus on the optimal surgical management for SMH or the key factors determining the outcome [7, 10].

In the absence of comparative studies, the aim of this study was to compare the structural and functional outcome after vitrectomy combined with subretinal rtPA, pneumatic displacement with or without an additional intravitreal injection of Bevacizumab under real-life conditions in a single vitreoretinal centre.

## Methods

Retrospective study of submacular hemorrhage patients treated with sutureless 23-gauge pars plana vitrectomy combined with a subretinal injection of tPA and pneumatic displacement with air tamponade between March 2018 and October 2019 in a single vitreoretinal surgery center (Eye Clinic Sulzbach, Knappschaft Hospital Saar, Germany) by 2 experienced vitreoretinal surgeons. This study was approved by the local ethics committee (Ethics committee Medical Association of Saarland, 252/15) and followed the declaration of Helsinki. Written informed consent was obtained.

Patients who met the following criteria were included in the study: vision impairment, acute onset of combined foveal subretinal and sub pigment epithelial hemorrhage verified by spectral domain optical coherence tomography (SD‑OCT, Heidelberg Engineering, Heidelberg, Germany) due to AMD [13, 14]. Acute onset was defined as subjective reduction in visual acuity within 1 week. To make the groups more homogeneous, only patients with a mean hemorrhage volume (MHV) between 10-20 mm^3^ were evaluated. Exclusion criteria were other aetiologies of SMH and phakic eyes, to exclude biased visual acuity values due to postoperative cataract formation [13]. Also, since isolated sub-pigment epithelial hemorrhages do not benefit from subretinal tPA treatment [14] they were excluded from the study.

All eyes were treated on the day of emergency presentation with 50 μg intravitreal tPA (Actilyse®, Boehringer Ingelheim, Germany) injection (dissolved in 0.05 ml Balanced salt solution (BSS)) and the following day with sutureless 23-gauge pars plana vitrectomy and received a subretinal 10μg tPA injection (dissolved in 0.1 ml BSS through a 41-gauge microcannula (DORC, Netherlands). A tamponade with air was applied. Postoperativly, patients were asked to maintain face position down. At the end of the surgery 14 patients received no intravitreal injection of 1.25 mg Bevacizumab (Avastin®, Roche, Switzerland) (group -B), whereas the 17 patients received an intravitreal Bevacizumab injection at the end of the surgery (group +B). 29/31 patients received further treatment with anti-VEGF injections postoperatively, at the earliest 4 weeks after surgery. The 2 remaining patients from group +B, who did not receive any further therapy, were not within the indication spectrum due to poor visual acuity.

At baseline and follow-up visits (4-6 weeks, 3 months), a slit lamp examination of the anterior segment, dilated funduscopy, OCT (optical coherence tomography), fluorescein angiography (FA), applanation tonometry and decimal best corrected visual acuity (BCVA,converted to logMAR for statistical analysis) were performed. Primary study endpoints were visual acuity (BCVA) and central macular thickness (CMT, in μm) measured with SD‑OCT (Spectralis® OCT (Heidelberg Engineering, Heidelberg, Germany). Secondary study endpoint was complete hemorrhage displacement, defined as free subfoveal space within 2.5 mm diameter around the foveal centre on Spectralis® SD-OCT scan and Spectralis® infrared fundus image [13]. To determine height and extension of the hemorrhages in relation to the RPE we used a spectral domain OCT (Spectralis®, Heidelberg engineering, Heidelberg, Germany). We performed a routine OCT volume scan of the macula region with a 30x30° pattern size (19 B-Scans with a distance of 235–240 μm (512 pixel x 496 pixel)) [15]. If needed the location of the fovea was adjusted manually. Three foveal images were analyzed including the central foveal scan and the upper and lower foveal region scan [14]. We did not use any additional software installation to display real-time treatment decisions. It is not always possible to obtain high-quality pictures on SD-OCT in patients with massive hemorrhages and automated contour-based image analysis could not work. Therefore, two investigators independently performed manual measurements on three OCT imagesThe Mean maximal hemorrhage diameter (MHD) was measured on the Spectralis infrared fundus image with the software’s ruler tool. In addition, if available and clinically indicated, MHD was determined during fluorescein angiography on the Spectralis HRA Blue Reflectance Red-free image (Heidelberg Engineering, Heidelberg, Germany). CMT was measured on the OCT image with the software’s ruler tool. The measurement was performed from the internal limiting membrane to the choroidal side of the RPE (between the RPE and the Bruch’s membrane) [13, 14].

Statistical analysis was performed with R, version 3.6.3, and the lme4 package (Version 1.1-23) for model fitting. To test the effectiveness of the treatment, we used a mixed model approach. To further investigate these results, we derived estimated marginal means from the mixed model and conducted post-hoc tests to compare points in time with each other. A comparative statistical evaluation of pre- and postsurgical data was performed with a Linear Model ANOVA. To test whether the frequency distribution of a categorical variable differs from a theoretically assumed distribution, a Pearson’s chi-squared test was performed. The results are presented as arithmetic mean and standard deviation (± SD) for all examined groups. A p-value <0.05 was defined as statistically significant.

## Results

A total of 31 eyes of 31 patients met all inclusion criteria and were included in this retrospective study. The first 14 patients received no intravitreal injection of 1.25 mg Bevacizumab at the end of the surgery (group -B), whereas the following 17 patients received an intravitreal Bevacizumab injection at the end of the surgery (group +B). The preoperative characteristics are summarized in Table 1. To evaluate the postoperative benefit, we compared the functional and anatomical outcomes of the two groups after surgery at a final follow-up after 3 months (Table 2).

**Table 1:** Preoperative characteristics in eyes treated with vitrectomy, air tamponade and either subretinal tPA and simultaneous intravitreal Bevacizumab or subretinal tPA alone.

Variable	tPA + Bevacizumab	tPA	P-value
	n=17	n=14	
	(group +B)	(group -B)	
Age in years	81.7 ± 5.2	83.4 ± 4.6	0.21^2^
Sex (female/male)	65% / 35%	71% / 29%	0.72^1^
Mean duration of acute symptoms in days	3.3 ± 1.6	3.4 ± 1.5	0.49^2^
Eyes naive to treatment	1/17	0/14	0.79^1^
Mean preceeding anti-VEGF injections	6.5 ± 5.8	6.2 ± 6.1	0.22^2^
Anticoagulation or antiplatelet therapy	15/17	13/14	0.84^1^
Mean hemorrhage volume (MHV) in mm^3^	11.78 ± 3.04	14.75 ± 3.98	0.057^2^
Mean maximal hemorrhage diameter (MHD) in μm	5212 ± 1891	5983 ± 2112	0.18^2^

^1^Pearson’s Chi-squared test

^2^Linear Model ANOVA.

**Table 2:** Postoperative outcome 3 months after vitrectomy and air tamponade and either subretinal tPA and simultaneous intravitreal Bevacizumab or subretinal tPA alone.

*Variable*	*tPA + Bevacizumab*	*tPA*	*P-value*
	*n=17*	*n=14*	
	*(group +B)*	*(group -B)*	
***Mean Visual acuity in logMAR***			
Preoperative	1.37 ± 0.39	1.48 ± 0.48	0.96^1^
Postoperative	1.03 ± 0.57	1.01 ± 0.38	>0.99^1^
***Visual improvement***	65%	71%	0.35^2^
***Mean central macular thickness (CMT) in μm***			
Preoperative	607 ± 179	722 ± 216	0.054^1^
Postoperative	423 ± 205	460 ± 202	0.99^1^
***Foveal hemorrhage displacement***	47%	50%	0.44^2^

^1^Linear Model ANOVA

^2^ Pearson’s Chi-squared test.

**Fig 1:**
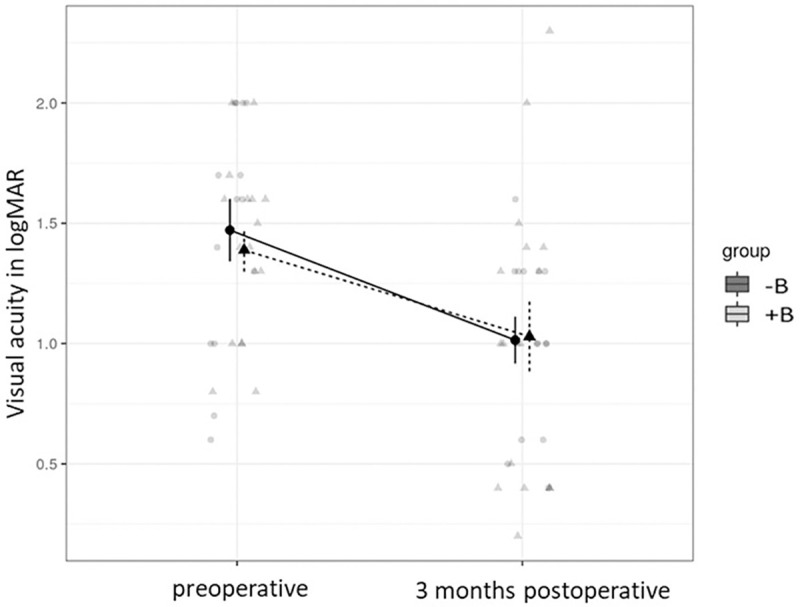
The mean visual acuity of the two groups (with (+) and without (-) Bevacizumab (B)). According to marginal means of visual acuity, group -B (1.48 ± 0.48 logMAR) did not differ from group +B, (1.37 ± 0.39 logMAR) before the surgery (p = 0.961). 3 months after surgery group -B (1.01 ± 0.38 logMAR) did not differ from group +B (1.03 ± 0.57 logMAR) (p>0.99). However, differences from pre- to postoperative visual acuity within the groups were significant for group -B (p = 0.005), as well as for group +B (p = 0.018) (Mixed Model, tukey moethod comparison).

The mean CMT of group -B (722 ± 216μm) did not differ from group +B (607 ± 179μm) before the surgery (p = 0.054), but group +B had a lower overall macular thickness. As well, group -B (460 ± 202μm) did not differ from group +B (423 ± 205μm) after the surgery (p = 0.99). However, differences in macular thickness from pre- to postoperative were significant for group -B (p<0.001) but not for group +B (p = 0.205). The mean visual acuity in both groups increased significantly (-B: p = 0.005 and +B: p = 0.018) after 3 months (Fig 1). We did not find an effect of group assignment (b = −0.07 95% CI[−0.42,0.25], p = 0.669). In addition, we did not find an interaction effect (b = 0.10 95% CI[−0.22,0.39], p = 0.589), meaning that postoperatively the effect did not differ between the two groups.

After 3 months, a foveal displacement of the hemorrhage could be achieved in 47% in group +B, whereas in group -B a displacement of 50% could be detected (p = 0.44). Likewise, a visual improvement of at least 1 line was found in 71% in group -B, compared to group +B with 65% after 3 months (p = 0.35).

Postoperative complications were found in three eyes in group +B. One eye had to undergo further surgery due to a macular hole, which was most likely caused by the intraoperative manipulation. Two eyes had a peripheral retinal detachment and needed further surgical intervention. No eyes showed a recurrence of SMH in group +B. Whereas one eye (1/14, 7%) in the group -B showed a recurrence of SMH after 4 weeks. No other complications were found in group -B. 29/31 patients received further treatment with anti-VEGF injections postoperatively, at the earliest 4 weeks after surgery. The 2 remaining patients from group +B, who did not receive any further therapy, were not within the indication spectrum due to poor visual acuity. We could not find any association with anticoagulation or antiplatelet medication and the rate of re-bleeding. A post-hoc analysis of the clinical outcome revealed no statistical correlation to the surgeon among the two groups.

## Discussion

To the best of our knowledge, this is the first study that evaluated the efficacy of subretinal rtPA treatment while differentiating between patients receiving intravitreal Bevacizumab or no additional treatment. Our results confirm the efficacy of subretinal rtPA therapy in a real-life clinical setting [9, 14, 16–19]. Although, the postoperative comparison of the two groups showed comparable values regardless of the intraoperative administration of bevacizumab into the vitreous or without. This could be due, amongst other reasons, to the fact that the subgroups were not fully comparable in terms of CMT and MHV: the group without bevacizumab had larger blood volumes preoperatively. Nevertheless, the functional outcome depends significantly on the extent of the underlying CNV and the progressive degenerative process of neovascular AMD [10]. Therefore, the application of bevacizumab intravitreally instead of subretinally, directly at the site of CNV, during vitrectomy could be another reason for our results.

The best approach in SMH continues to be discussed and large prospective randomized trials are still lacking but planned (TIGER-study). Meyer at al. have already reported that the intravitreal application of rtPA, gas and bevacizumab appears to be beneficial and seems logical in limiting the progression of the underlying disease [19]. Even if Guthoff et al. could show that there is a strong indication that the addition of intravitreal bevacizumab is superior to the displacement of submacular hemorrhages [20], we could not confirm this in our study. However, while the administration of rtPA can prevent a toxic effect of the blood by displacing the SMH from the fovea, the simultaneous administration of an anti-VEGF agent could affect the progression of CNV [21]. However, it could be possible that proteases activated during fibrinolysis cleave bevacizumab intraoperatively during co-administration and thus functionally inactivate it. But studies could disprove this and showed no cleavage or functional inactivation of bevacizumab, when given alongside tPA in an in vitro model [21–23]. Furthermore, simultaneous application of 25 mg/mL bevacizumab and 20 mg/mL tPA also produced no sign of retinal toxicity on electroretinography [24].

Even if Bevacizumab (149 kDa) exceeds the transretinal diffusion limit under normal conditions [25], the theoretical retinal exclusion limit is not absolute, and larger molecules will still traverse slowly the retina, possibly leading to a longer drug retention and sustained duration of action [25, 26]. But an SMH could also impede transretinal diffusion, and therefore it is conceivable that intravitreal bevacizumab do not reach the CNV underlying the haemorrhage in sufficient concentration and quantity [21]. This could be also an explanation for our results.

Thus, while it is uncertain whether bevacizumab after intravitreal injection is sufficiently therapeutically effective, the alternative technique of combined subretinal injection allows direct dissolution of the clotted SRH by tPA and application of bevacizumab directly at the CNV. In addition, this could potentially enhance the anti-angiogenic effect of bevacizumab [17, 21]. Supporting this, Treumer et al. reported short term and longterm follow-up of SMH treated by vitrectomy and subretinal co-application of both bevacizumab and tPA, followed by fluid-air exchange and SF6 gas, and subsequent intravitreal bevacizumab, using an as needed dosing regimen. They could not show clinical signs of retinal toxicity such as geographic atrophy or retinal degeneration [10, 17, 27]. Nethertheless, it should be considered that a subretinal injection implicates a possible risk of damaging the RPE, especially with underlying PED [10]. Therefore, we had decided against subretinal bevacizumab application before the study. However, Treumer et al. had a RPE rip rate of 9%, which is comparable to the risk after intravitreal injection [10]. Even if we could not show any RPE rip in our study, the observed complications in this study were in consistency with those described in other studies [8, 9, 14, 17].

It can be assumed that an additional intraoperative application of bevacizumab could positively influence the recurrence rate of SRH. Indeed, SRH did not reoccur in the Bevacizumab group. While one eye (7%) in the group without Bevacizumab had a recurrence of SRH. Therefore, we could show a much lower recurrence rate than described in literature with 20-29% [10, 28]. However, these recurrences were detected several months postoperatively while our study is limited due to a short follow-up of 3 months. Further limitation of this study was the non-randomized retrospective study design with a rather small and inhomogeneous group size, despite restriction of the inclusion criteria.

In conclusion, vitrectomy with subretinal tPA injection and air tamponade is effective in the treatment of subretinal hemorrhages due to AMD. But in comparison, the postoperative outcome is comparable regardless of whether or not intravitreal bevacizumab is applied simultaneously. Further comparative randomised trials, especially between intravitreal and subretinal application of bevacizumab, would be of further interest.
